# Endovascular Therapy for Acute Basilar Artery Occlusion: Prognosis Prediction Value from Clinical to Imaging Variables

**DOI:** 10.31083/RN37034

**Published:** 2025-10-21

**Authors:** Shunyang Chen, Yiying Pan, Pengjun Chen, Chenchen Hong, Tian Gao, Chaoming Huang, Jiansong Ji

**Affiliations:** ^1^Department of Radiology, Lishui Central Hospital, Shaoxing University, 312000 Shaoxing, Zhejiang, China; ^2^Department of Radiology, Zhejiang Cancer Hospital, Hangzhou Institute of Medicine (HIM), Chinese Academy of Sciences, 310022 Hangzhou, Zhejiang, China; ^3^Zhejiang Provincial Key Laboratory of Imaging Diagnosis and Minimally Invasive Intervention Research, The Fifth Affiliated Hospital of Wenzhou Medical University, Lishui Hospital of Zhejiang University, 323000 Lishui, Zhejiang, China

**Keywords:** prognosis, basilar artery occlusion, endovascular therapy, stroke, pronóstico, oclusión de la arteria basilar, terapia endovascular, accidente cerebrovascular

## Abstract

**Purpose:**

Acute basilar artery occlusion (BAO) correlates with high risks of disability and mortality, and the best imaging and treatment strategies for BAO remain controversial. This study evaluated the association between baseline imaging, clinical variables, and clinical outcomes of patients with BAO undergoing endovascular therapy (EVT).

**Methods:**

Data from 75 patients with BAO who had EVT at a single center were retrospectively analyzed. Baseline National Institutes of Health Stroke Scale (NIHSS) scores, clinical baseline data, and various known scores and perfusion deficit volumes on non-contrast computed tomography (NCCT), CT angiography source images (CTA-SI), and CT perfusion (CTP) were collected to explore effective predictive factors for prognosis. The functional outcome of the analysis was satisfactory (90-day modified Rankin Scale score ≤3). Predictors of functional outcomes were assessed through receiver operating characteristic analyses and binary logistic regression.

**Results:**

Among the 75 patients who fulfilled the inclusion criteria, 29 achieved a good outcome (39%) and 46 (61%) achieved a poor outcome. The Critical Area Perfusion Score (CAPS), pons midbrain index (PMI), time to maximum (Tmax) >6 s, Tmax >10 s, and reduction in CBF compared with normal brain tissue (rCBF) <30%, cerebral blood flow (CBF), cerebral blood volume (CBV), and mean transit time (MTT) Posterior Circulation Alberta Stroke Program Early CT Score (pc-ASPECTS) were independent predictors of favorable prognosis. The CAPS was the best predictor of good clinical outcomes, with an area under the curve of 0.862 (95% confidence interval [CI], 0.772–0.952). Combined diagnosis with the baseline NIHSS score improved the prognosis prediction accuracy.

**Conclusions:**

In patients with stroke that resulted in BAO after EVT, CAPS, PMI, Tmax >6 s, Tmax >10 s, rCBF <30% volume, and CBV pc-ASPECTS were excellent predictors of higher risk of disability and mortality. Furthermore, CAPS had the best accuracy, and overall predictive value could be improved when combined with the baseline NIHSS score for diagnosis.

## Abstract (es)

**Objetivo:**

La oclusión aguda de la arteria basilar (OAB) se correlaciona con un alto riesgo de discapacidad y mortalidad, y las mejores estrategias de diagnóstico por la imagen y tratamiento para la OAB siguen siendo controvertidas. Este estudio evaluó la asociación entre las imágenes iniciales, las variables clínicas y los resultados clínicos de pacientes con OAB sometidos a tratamiento endovascular (TEV).

**Métodos:**

Se analizaron retrospectivamente los datos de 75 pacientes con OAB que se sometieron a TEV en un único centro. Se recopilaron las puntuaciones iniciales de la escala del accidente cerebrovascular del National Institutes of Health (NIHSS), los datos clínicos iniciales y diversas puntuaciones conocidas y volúmenes de déficit de perfusión en tomografías computarizadas sin contraste (NCCT), imágenes de angiografía por TC (CTA-SI) y perfusión por TC (CTP) para explorar los factores predictivos eficaces para el pronóstico. El resultado funcional del análisis fue satisfactorio (puntuación en la escala de Rankin modificada a los 90 días ≤3). Los predictores de los resultados funcionales se evaluaron mediante análisis de características operativas del receptor y regresión logística binaria.

**Resultados:**

De los 75 pacientes que cumplieron los criterios de inclusión, 29 obtuvieron un buen resultado (39%) y 46 (61%) obtuvieron un mal resultado. La puntuación de perfusión del área crítica (CAPS, *Critical Area Perfusion Score*), el índice puente-mesencéfalo (PMI, *pons midbrain index*), el tiempo hasta el máximo (Tmáx) >6 s, Tmáx >10 s y reducción del FSC en comparación con el tejido cerebral normal (rFSC) <30%, el flujo sanguíneo cerebral (FSC), el volumen sanguíneo cerebral (VSC) y el tiempo de tránsito medio (TTM) de la puntuación del programa de Alberta para el accidente cerebrovascular en la circulación posterior (pc-ASPECTS, *Posterior Circulation Alberta Stroke Program Early CT Score*) fueron predictores independientes de un pronóstico favorable. El CAPS fue el mejor predictor de buenos resultados clínicos, con un área bajo la curva de 0,862 (intervalo de confianza [IC] del 95%, 0,772–0,952). El diagnóstico combinado con la puntuación NIHSS inicial mejoró la precisión de la predicción del pronóstico.

**Conclusiones:**

En pacientes con accidente cerebrovascular que provocó una OAB tras una TEV, el CAPS, el PMI, el Tmáx >6 s, el Tmáx >10 s, el rFSC <30% del volumen y el VSC pc-ASPECTS fueron excelentes predictores de un mayor riesgo de discapacidad y mortalidad. Además, el CAPS tuvo la mejor precisión, y el valor predictivo global pudo mejorarse cuando se combinó con la puntuación NIHSS inicial para el diagnóstico.

## 1. Introduction

The basilar artery is the principal artery in the posterior circulation and the central component of the vascular area [1]. Basilar artery occlusion (BAO) is the most destructive subtype of stroke, with clinical signs varying from modest transitory symptoms to severe stroke. BAO accounts for 10% of all large vessel occlusions (LVO) and approximately 1% of all ischemic strokes [2]. Mortality or disability affects almost 80% of people with BAO who do not receive treatment [3, 4]. While the ideal therapeutic approach for BAO continues to be contentious, endovascular therapy (EVT) is currently recommended for anterior circulation ischemic stroke (ACIS) [5, 6, 7]; however, its safety and efficacy in the posterior circulation remain unclear. Recent randomized trials, including the Trial of Endovascular Treatment of Acute Basilar-Artery Occlusion (ATTENTION) [8], Acute Basilar Artery Occlusion Study (BASILAR) [9], and the Basilar Artery Occlusion Chinese Endovascular trials (BAOCHE) [10], have found a greater percentage of patients with a favorable prognosis at 3 months after EVT than medical treatment in patients with BAO, indicating that BAO might benefit from EVT. Therefore, it is essential to accurately diagnose and evaluate BAO at an early stage to identify patients who are likely to derive advantage from preoperative treatment and to avoid ineffective therapies [11]. However, predictive factors for this phenomenon require further investigation. Appropriate imaging may help predict prognosis in patients with BAO [12, 13].

Under valid preoperative imaging screening criteria, the BAOCHE trial [10] discovered the potential for EVT benefits in patients with BAO. Non-contrast computed tomography (NCCT) is the predominant diagnostic technique for stroke, which helps to quickly rule out hemorrhagic cerebral infarction or other diseases [8]. However, NCCT exhibits lower specificity for posterior circulation and can detect only about 20–40% of patients in the early stages of posterior circulation ischemia [14]. CT angiography (CTA) is commonly used to detect vascular occlusion. CTA source images (CTA-SI) can identify ischemic brain tissue, aiding the early detection of ischemic changes in the brain tissue of patients with acute stroke. Previous studies have proposed a semi-quantitative scoring system based on CTA imaging to quantify the extent of posterior circulation vascular occlusion, including pons-midbrain and thalamus (PMI) [15], posterior circulation collateral score (pc-CS) [16], posterior circulation CTA (pc-CTA) [17] score, and basilar artery CTA prognosis score (BATMAN) [18].

CT perfusion (CTP) images have been shown to provide significant predictive value in patients with ischemic stroke in the anterior circulation [19]. Puetz* et al*. [20] proposed the Posterior Circulation Alberta Stroke Program Early CT Score (pc-ASPECTS) score to evaluate ischemic stroke in the posterior circulation. As imaging technology evolves, research has found that the CTP image-based pc-ASPECTS score has excellent application value compared to that on the basis of NCCT or CTA-SI and has a higher value in detecting ultra-early stroke and predicting long-term postoperative outcomes. Cereda *et al*. [21] first defined the Critical Area Perfusion Score (CAPS) on time to maximum (Tmax) >10 s maps of perfusion imaging and achieved the ideal predictive power compared to other predictors. However, its use as a forecasting tool still lacks external validation. In addition to various imaging scores, such as the DAWN [22] and DEFUSE [23], studies indicate that ischemic stroke in the anterior circulation can be evaluated from perfusion deficit volume to assess the extent to which patients may benefit from EVT. However, there are few studies on BAO, and the optimal threshold has not been established.

The Basilar Artery International Cooperation Study (BASICS) recommends EVT for BAO with a baseline NIHSS score >10. However, it is unknown whether medical or EVT treatment is recommended for patients with a baseline NIHSS score <10 [24]. Previous studies have shown the utility of EVT in ischemic stroke in the posterior circulation, and the baseline NIHSS score was a significant and independent predictor of prognosis in patients after 3 months [25, 26]. However, it is inherently biased towards motor and cortical deficits associated with ACIS; therefore, combined emergency imaging evaluation is necessary to improve diagnostic sensitivity and accuracy, help formulate reasonable treatment strategies, and evaluate prognosis.

## 2. Methods

### 2.1 Study Population

This study was approved by the Institutional Review Board of Lishui Central Hospital (approval number 2024073), and the requirement for written informed consent was waived. We retrospectively analyzed patients with BAO who accepted a multimodal CT examination before EVT between April 2018 and August 2023. Intravenous thrombolysis is allowed before EVT according to present guidelines, and patients had to be treated within 24 hours of onset. Multimodal CT examination included NCCT, CTP, and CTA. Imaging follow-up was done within 1–3 days post-reperfusion therapy.

**Fig. 1.**
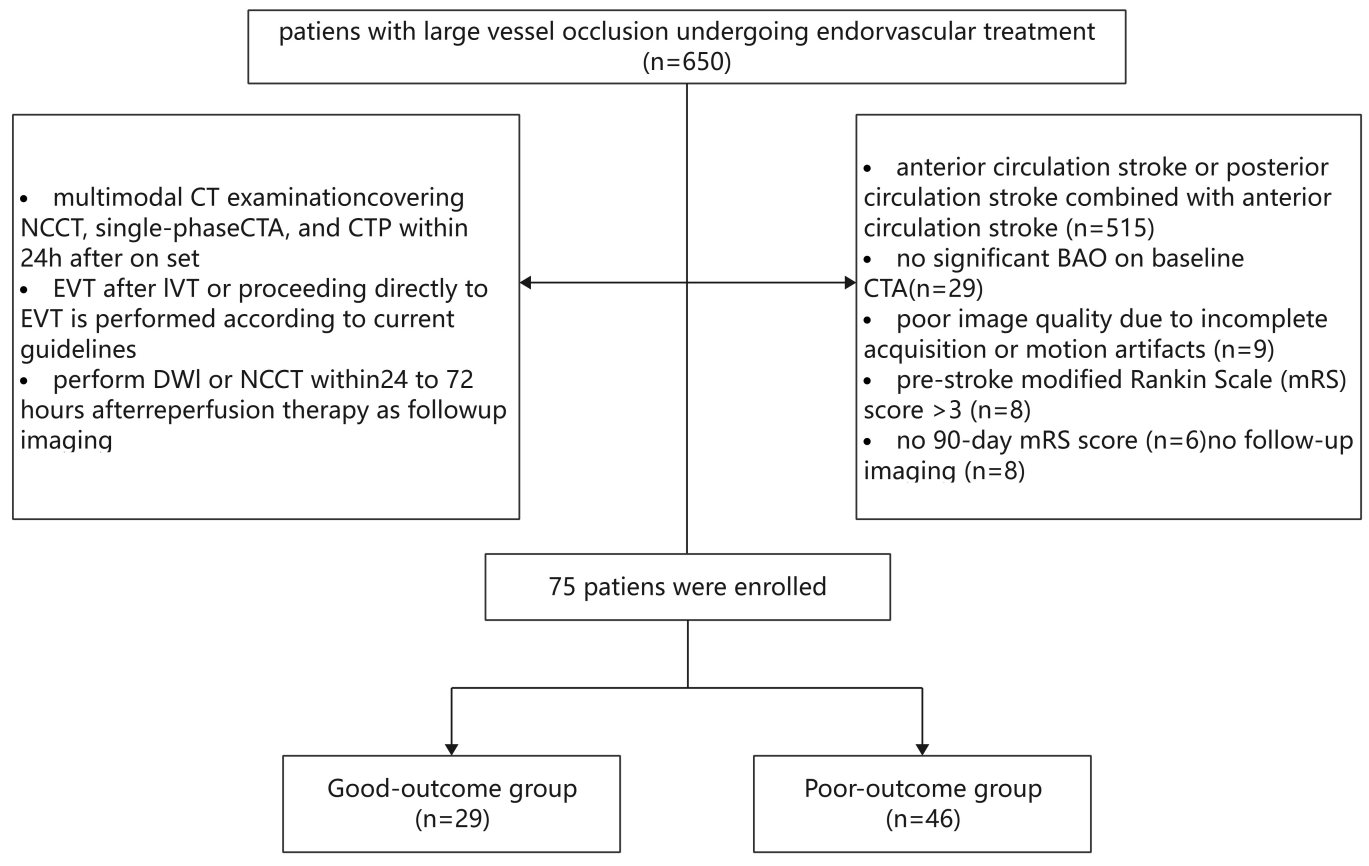
**Enrollment flowchart**. NCCT, non-contrast computed tomography; CTA, CT angiography; CTP, CT perfusion; EVT, endovascular therapy; BAO, basilar artery occlusion; IVT, intracranial venous thrombolysis; DWI, diffusion-weighted imaging.

Overall, 575 patients were excluded because they had (a) anterior circulatory stroke (n = 515); (b) a pre-stroke modified Rankin Scale (mRS) score >3 (n = 8); (c) no 90-day mRS score (n = 6); (d) no follow-up scanning (n = 8); (e) poor image quality (n = 9); or (f) baseline CTA failed to confirm BAO (n = 29) [16, 27] (Fig. 1). According to prior research, a good outcome is indicated by an mRS score of ≤3 at 90 days post-stroke [28].

### 2.2 Clinical Data

Clinical data, including sex, age, baseline NIHSS score, coma (defined as a Glasgow Coma Scale [GCS] ≤8), risk factors, mRS scores, onset-to-treatment time (OTT), onset-to-scan time (OST), etiology, and treatment data, were collected by trained investigators who were blinded to outcomes of interest and imaging data.

### 2.3 Multiparametric CT Imaging and Analysis

Patients received a standardized multiparametric CT procedure that included noncontrast CT, single-phase CTA, and whole-brain CTP, conducted upon admission using Siemens Syngo.via CT Neuro Perfusion version VB40 (Siemens Healthcare, Erlangen, Germany). After a preliminary 4-second delay, 50 mL of contrast was administered at a rate of 5 mL/s, and perfusion of volume for 51 seconds. CT data was gathered every 1.5 to 3 seconds. Slides were rebuilt for the perfusion analysis using a thickness of 5 mm every 3 mm and for CTA analysis using a thickness of 0.625 mm every 1 mm. Scanning WB-CTP data were sent to Syngo.via, an automated program to return mean transit time (MTT), cerebral blood flow (CBF), cerebral blood volume (CBV), and Tmax maps, as well as volume of CT perfusion deficit with predefined criteria. Using pc-ASPECTS [19, 29, 30], our research assessed CTP, CTA-SI, and NCCT scans to capture early ischemia alterations in patients quickly. That matched the CAPS allocation shown in previous studies. Following brain areas: cerebellum (1 point per hemisphere), pons (2 points), or midbrain or thalamus (2 points), CAPS was awarded according to the occurrence of a Tmax >10-second delay [21].

The PMI [15], Basilar Artery on Computed Tomography Angiography (BATMAN) [18], posterior circulation CTA (pc-CTA) [17], and pc-CS [16], were evaluated using CTA while simultaneously recording vessel occlusion and cerebral atherosclerosis. In addition, potential recuperation ratio, mismatch volume, reduction in CBF compared with normal brain tissue (rCBF) below 20% and 30% volume, and Tmax exceeding 6- and 10-second volumes were collected using Syngo.via.

**Fig. 2.**
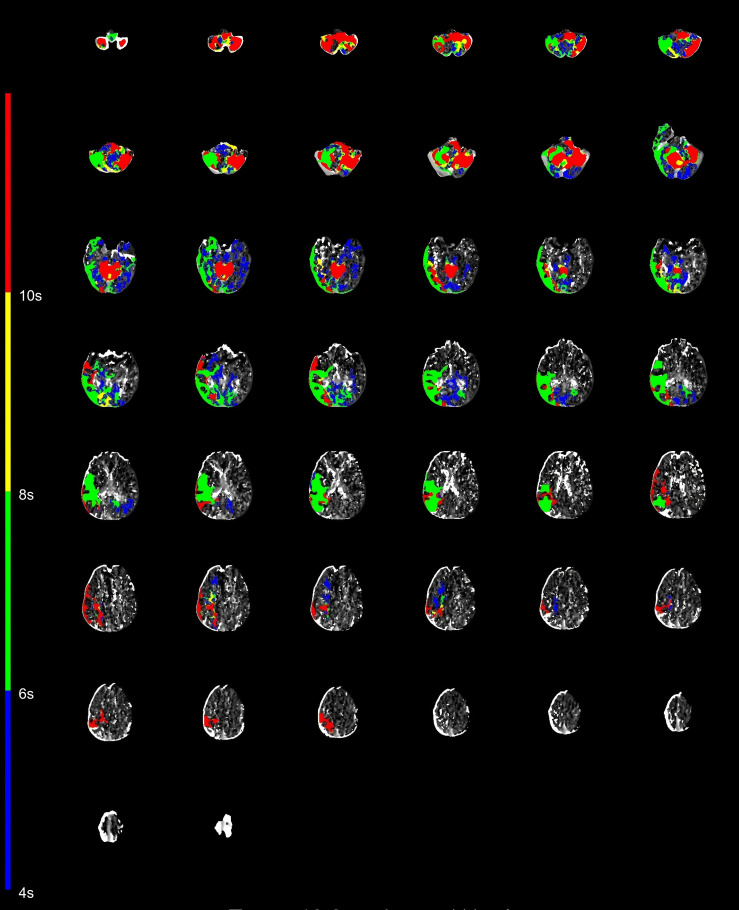
**Case example of CAPS**. Images obtained from a 75-year-old male patient with a CAPS score of 5 (midbrain/thalamus, 2 points; pons, 2 points; and complete cerebellar hemisphere, 1 point). CAPS, Critical Area Perfusion Score.

**Fig. 3.**
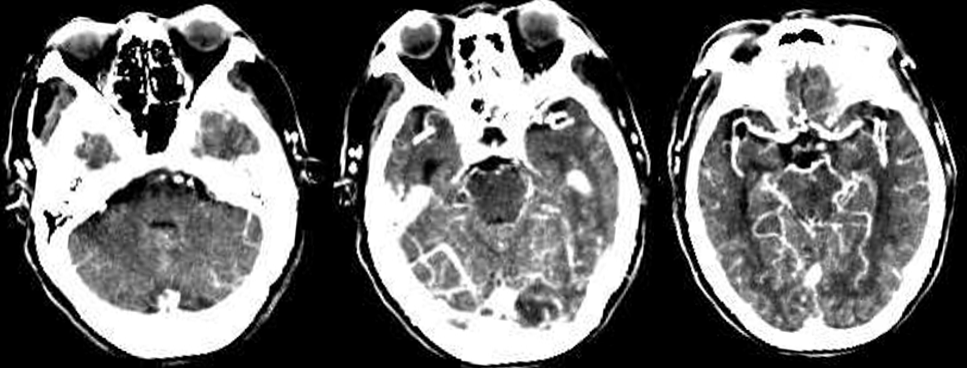
**Case example of PMI**. Images obtained from a 56-year-old female patient with a PMI score of 7 (right pons, 2 points, left pons, 2 points, left midbrain, 2 points, and right midbrain, 1 point). PMI, pons midbrain index.

The images were independently assessed by two investigators using a double-blind approach to prevent them from identifying the prognosis and subsequent scanning consequence. In the event of disagreement, consensus was established in another meeting. Figs. 2,3 illustrates examples.

### 2.4 Statistical Analysis

Statistical analyses were conducted using SPSS (SPSS 23; IBM, Armonk, NY, USA). Continuous variables were expressed as medians and interquartile ranges (IQR) and compared using the Mann–Whitney U test. Univariable analysis was performed using the Mann–Whitney U test for continuous variables and the Pearson χ^2^ or Fisher exact test for categorical variables to compare baseline and imaging characteristics between patients with good and poor outcomes. Candidate variables (*p* values < 0.1) in the univariable analysis were included in the multivariable logistic regression model. Collinearity was diagnosed using variance inflation factors (VIF) in multivariable logistic regression, and multivariable logistic regression analysis was used to identify independent predictors of favorable outcomes. The prognostic performance of variables was evaluated by receiver operating characteristic (ROC) curve and area under the curve (AUC) analyses. Youden techniques were used to determine the optimal cut-off value. A *p* < 0.05 (two-sided) was considered to indicate statistical significance.

## 3. Results

### 3.1 Patient Characteristics

A total of 650 patients were included, of whom 75 patients with BAO received EVT. A total of 29 cases (39%) had good outcomes, and 46 cases (61%) had poor outcomes at the 90-day follow-up. The rates of 90-day mortality (n = 21, 28.0%) and survival (n = 54, 72.0%) were compared. The baseline data of patients with acute BAO with good versus poor clinical outcomes and survival versus non-survival are displayed in Tables 1,2, respectively. Patients had average baseline NIHSS scores of 6–40, and 61 patients presented with coma upon admission.

**Table 1.** **Baseline characteristics of patients with acute BAO**.

Baseline variables		All patients (n = 75)	Good-outcome group (n = 29)	Poor-outcome group (n = 46)	*p*-value
Men, n (%)		51 (68.0)	20 (69.0)	31 (67.4)	0.887
Age, y, median (IQR)		68 ± 10	66 ± 10	70 ± 10	0.792
Onset-to-scan time (min)		401 (280–510)	389 (240–563)	422 (329–483)	0.418
Coma		61 (81.3)	17 (58.6)	44 (95.7)	<0.001*
Baseline NIHSS score		26 (18–30)	15 (10–19)	28 (26–32)	<0.001*
Premorbid mRS score		0 (0-0)	0 (0–0)	0 (0–0)	0.422
90-d mRS score		4 (1–6)	1 (0–2)	6 (4–6)	<0.001*
Treatment data					
	Intravenous thrombolysis	15 (20.0)	9 (31.0)	6 (13.0)	0.058
	Onset-to-treatment time (min)	495 (383–624)	492 (356–722)	498 (405–605)	0.373
Risk factors					
	Arterial hypertension	59 (78.7)	20 (76.9)	30 (78.9)	1.000
	Diabetes mellitus	30 (40.0)	8 (27.6)	21 (45.7)	0.081
	Hyperlipidemia	12 (16.0)	2 (6.8)	10 (21.7)	0.088
	Atrial fibrillation	12 (16.0)	5 (17.2)	7 (15.2)	0.816
	Coronary artery disease	8 (10.7)	3 (10.3)	5 (10.9)	0.943
	Prior cerebrovascular accident	27 (36.0)	9 (31.0)	18 (39.1)	0.477
	Alcohol intake	10 (12.5)	5 (17.2)	5 (10.9)	0.429
	Smoking history	22 (29.3)	9 (31.0)	13 (28.3)	0.797
Etiology of stroke					
	Cardioembolic	10 (13.3)	5 (17.2)	5 (10.9)	0.429
	Large artery atherosclerosis	38 (50.7)	14 (48.3)	24 (52.2)	0.422
	Other determined	10 (13.3)	6 (20.7)	4 (8.7)	0.137
	Undetermined	17 (22.7)	5 (17.2)	12 (26.1)	0.373
Baseline imaging					
	BATMAN	4 ± 2	4 ± 2	3 ± 2	0.003*
	pc-CTA score	3 (2–4)	3 (2–4)	2 (1–3)	0.023*
	pc-CS score	4 (3–6)	5 (3–7)	4 (2–5)	0.076
	Cerebral atherosclerosis	59 (78.7)	23 (79.3)	36 (78.2)	0.422
	PMI	3 (2–5)	2 (1–3)	4 (3–6)	<0.001*
CTP & pc-ASPECTS					
	NCCT pc-ASPECTS	9 (7–10)	9 (8–10)	8 (6–9)	0.025*
	CTA-SI pc-ASPECTS	8 (6–10)	9 (8–10)	7 (5–9)	0.026*
	CBF pc-ASPECTS	6 (6–7)	5 (4–6)	7 (5–8)	<0.001*
	CBV pc-ASPECTS	8 (5–9)	8 (8–10)	7 (6–8)	<0.001*
	MTT pc-ASPECTS	6 (3–7)	7 (6–8)	4 (3–6)	<0.001*
	Tmax pc-ASPECTS	3 (2–6)	5 (3–7)	2 (2–4)	0.012*
	CAPS (Tmax >10 s)	3 (2–4)	2 (1–3)	4 (3–5)	0.001*
RAPID pc-ASPECTS					
	Tmax >4 s pc-ASPECTS	8 (7–8)	7 (6–8)	7 (7–8)	0.142
	Tmax >6 s pc-ASPECTS	6 (5–7)	6 (5–7)	6 (6–7)	0.016*
	Tmax >8 s pc-ASPECTS	5 (5–6)	5 (4–6)	5 (5–6)	0.172
Perfusion deficit volume					
	Mismatch volume (mL)	81.29 (32.38–115.29)	57.30 (27.61–81.70)	96.42 (43.95–123.07)	0.007*
	PRR (%)	89.13 (85.99–100)	94.95 (93.26–100)	85.47 (41.05–98.64)	0.019*
	VTmax >6 s (mL)	89.33 (35.94–116.31)	50.99 (24.19–78.39)	111.22 (58.66–140.55)	<0.001*
	VTmax >10 s (mL)	14.68 (0–17.85)	2.29 (0–3.60)	22.22 (4.19–31.02)	<0.001*
	VrCBF <20% (mL)	4.11 (0–5.1)	2.01 (0–2.14)	5.75 (0–8.64)	0.018*
	VrCBF <30% (mL)	9.27 (0–13.4)	2.34 (0–2.56)	13.98 (2.29–20.43)	<0.001*
	Cerebellar tonsillar hernia	17 (22.7)	0 (0)	17 (37.0)	<0.001*
	Symptomatic intracranial haemorrhage	29 (38.7)	4 (13.8)	25 (54.3)	<0.001*
	PICA infarction	30 (36.6)	6 (20.7)	24 (52.2)	0.007*
	AICA infarction	34 (41.5)	7 (24.1)	27 (58.7)	0.003*
	SCA infarction	48 (58.5)	16 (55.2)	32 (69.6)	0.206
	Pons infarction	57 (69.5)	18 (62.1)	39 (63.0)	0.025*
	Midbrain infarction	39 (47.6)	8 (27.6)	31 (67.4)	0.001*
	Thalamus infarction	38 (46.3)	10 (34.5)	28 (60.9)	0.051
	PCA infarction	24 (29.3)	7 (24.1)	17 (37.0)	0.026*
Location of occlusion					
	Proximal basilar artery	42 (56.0)	13 (44.8)	30 (65.2)	0.082
	Middle basilar artery	54 (72.0)	25 (86.2)	33 (71.7)	0.816
	Distal basilar artery	46 (61.3)	16 (55.1)	39 (84.8)	0.005*
	Vertebral arteries	29 (38.7)	6 (20.7)	22 (47.8)	0.018*
	Posterior cerebral artery	47 (62.7)	15 (51.7)	33 (71.7)	0.046*

SCA, superior cerebellar artery; AICA, anterior inferior cerebellar artery; PICA, posterior inferior cerebellar artery; PCA, posterior cerebral artery; rCBV, reduction in CBF value compared with normal brain tissue; mismatch volume, VTmax >6 s-VrCBF <30%; Tmax, time-to-maximum deficit volume; potential recuperation ratio, mismatch volume/VTmax >6 s; MTT, mean transit time; CBV, cerebral blood volume; CBF, cerebral blood flow; pc-ASPECTS, posterior circulation-Alberta Stroke Program Early CT Score; CTA-SI, computed tomography angiography source image; NCCT, non-contrast CT; pc-CS, posterior circulation-collateral score; pc-CTA, posterior circulation-computed tomography angiography; NIHSS, National Institute of Health Stroke Scale; BATMAN, basilar artery on computed tomography angiography; Good-outcome Group, 90-day mRS score 0–3; Poor-outcome Group, 90-day mRS score 4–6. * indicates *p* < 0.05.

**Table 2.** **Baseline characteristics of patients with acute BAO**.

Baseline variables		Survival group (n = 54)	Non-survival group (n = 21)	*p*-value
Men, n (%)		24 (44.4)	12 (57.1)	0.209
Age, y, median (IQR)		68 ± 10	71 ± 11	0.568
Onset-to-scan time (min)		422 (268–452)	426 (346–488)	0.363
Coma		32 (59.2)	20 (95.2)	0.205
Baseline NIHSS score		27 (24–30)	28 (24–30)	0.013*
Premobid mRS score		0 (0–0)	0 (0–0)	0.622
90d mRS score		3 (2–5)	6 (6–6)	<0.001*
Treatment data				
	Intravenous thrombolysis	9 (16.7)	2 (9.5)	0.157
	Onset-to-treatment time (min)	513 (347–562)	507 (441–606)	0.109
Risk factors				
	Arterial hypertension	29 (53.7)	16 (76.2)	0.744
	Diabetes mellitus	12 (22.2)	12 (57.1)	0.172
	Hyperlipidemia	4 (7.4)	4 (19.0)	0.653
	Atrial fibrillation	9 (16.7)	1 (4.8)	0.098
	Coronary artery disease	4 (7.4)	2 (9.5)	0.842
	Prior cerebrovascular accident	10 (18.5)	11 (52.4)	0.191
	Alcohol intake	3 (5.6)	3 (14.3)	0.881
	Smoking history	11 (20.4)	3 (14.3)	0.074
Etiology of stroke				
	Cardioembolic	8 (14.8)	2 (9.5)	0.545
	Large artery atherosclerosis	15 (27.8)	12 (57.1)	0.484
	Other determined	3 (5.6)	1 (4.8)	0.173
	Undetermined	8 (14.8)	5 (23.8)	0.446
Baseline imaging				
	BATMAN	4 (2–5)	3 (2–5)	0.185
	pc-CTA score	3 (2–4)	2 (0–3)	0.043*
	pc-CS score	4 (3–6)	4 (2–5)	0.289
	Cerebral atherosclerosis	6 (11.1)	1 (4.8)	0.353
	PMI	3 (2–4)	5 (4–6)	<0.001*
CTP & pc-ASPECTS				
	NCCT pc-ASPECTS	8 (8–10)	8 (6–10)	0.323
	CTA-SI pc-ASPECTS	8 (7–10)	7 (5–10)	0.082
	CBF pc-ASPECTS	6 (5–8)	5 (4–6)	0.004*
	CBV pc-ASPECTS	8 (7–8)	6 (5–8)	0.024*
	MTT pc-ASPECTS	6 (3–7)	4 (3–6)	0.028*
	Tmax pc-ASPECTS	4 (2–6)	3 (2–4)	0.138
	CAPS (Tmax >10 s)	3 (1–4)	3 (3–4)	0.649
RAPID pc-ASPECTS				
	Tmax >4 s pc-ASPECTS	7 (6–8)	7 (7–8)	0.722
	Tmax >6 s pc-ASPECTS	6 (5–7)	6 (6–7)	0.608
	Tmax >8 s pc-ASPECTS	5 (4–6)	5 (5–6)	0.096
Perfusion deficit volume				
	Mismatch volume (mL)	77.46 (27.89–106.17)	91.15 (50.34–119.22)	0.101
	PRR (%)	91.28 (90.03–100.00)	83.61 (80.81–98.50)	0.103
	VTmax >6 s (mL)	84.65 (30.48–112.07)	101.36 (60.67–123.57)	0.049*
	VTmax >10 s (mL)	11.48 (0–12.50)	22.90 (5.48–32.44)	<0.001*
	VrCBF <20% (mL)	4.02 (0–5.33)	4.34 (0–4.69)	0.569
	VrCBF <30% (mL)	7.76 (0–10.23)	13.15 (2.33–19.87)	0.035*
	Cerebellar tonsillar hernia	7 (61.1)	10 (47.6)	0.001*
	Symptomatic intracranial hemorrhage	17 (51.5)	12 (57.1)	0.04*
	PICA infarction	19 (35.2)	11 (52.4)	0.172
	AICA infarction	20 (37.0)	14 (66.7)	0.021*
	SCA infarction	32 (59.2)	16 (76.2)	0.171
	Pons infarction	38 (70.4)	19 (90.5)	0.067
	Midbrain infarction	23 (42.6)	16 (76.2)	0.009*
	Thalamus infarction	24 (44.4)	13 (61.9)	0.174
	PCA infarction	20 (37.0)	10 (47.6)	0.401
Location of occlusion				
	Proximal basilar artery	29 (53.7)	13 (61.9)	0.521
	Middle basilar artery	44 (81.5)	18 (85.7)	0.664
	Distal basilar artery	37 (68.5)	18 (85.7)	0.131
	Vertebral arteries	16 (29.6)	11 (52.4)	0.065
	Posterior cerebral artery	33 (61.1)	14 (66.7)	0.655

Good-outcome Group, 90-day mRS score 0–3; Poor-outcome Group, 90-day mRS score 4–6. * indicates *p* < 0.05.

The patients with BAO with poor outcomes had a higher baseline NIHSS score (*p* < 0.001) and had more distal BAOs (*p* = 0.005), unilateral or bilateral vertebral artery occlusions (*p* = 0.018), and posterior cerebral artery occlusions (*p* = 0.046) than those with a good prognosis. The posterior inferior cerebellar artery (*p* = 0.007), midbrain (*p* < 0.001), pons (*p* = 0.025), and anterior inferior cerebellar artery (*p* = 0.003) were more probable for involvement. Cerebellar tonsillar hernia (*p* < 0.001) and hemorrhagic transformation (*p* < 0.001) were much more common. In contrast, there were no significant differences between the good-outcome and poor-outcome groups regarding pre-onset modified Rankin Scale score, risk factors, age, sex, etiology, and OST (*p* > 0.05 for all).

### 3.2 Prognostic Value of Baseline Imaging Parameters

Concerning‌ baseline images, PMI (*p* < 0.001) and CAPS scores (*p* < 0.001) were worse in the 90-day poor outcome group, and pc-ASPECTS scores were lower on NCCT (*p* = 0.025), CTA-SI (*p* = 0.026), CBF (*p* < 0.001), CBV (*p* < 0.001), MTT (*p* < 0.001), Tmax (*p* = 0.012), and RAPID Tmax >6 s (*p* = 0.016) images. The BATMAN score (*p* = 0.003) and pc-CTA (*p* = 0.023) were also associated with prognosis at 90 days.

In the multivariable binary logistic regression analyses, factors with *p* < 0.1, such as coma, intravenous thrombolysis, and diabetes mellitus, were adjusted. CAPS score (odds ratio [OR], 2.64; 95% confidence interval [CI], 1.700–4.097; *p* < 0.001), PMI (OR, 3.72; 95% CI, 1.264–10.962; *p* = 0.017), baseline NIHSS score (OR, 1.18; 95% CI, 1.076–1; *p* < 0.001), BATMAN scores (OR, 0.73; 95% CI, 0.558–0.942; *p* = 0.016), CBF pc-ASPECTS (OR, 0.62; 95% CI, 0.446–0.853; *p* = 0.003), CBV pc-ASPECTS (OR, 0.25; 95% CI, 0.077–0.804; *p* = 0.002), MTT pc-ASPECTS (OR, 0.69; 95% CI, 0.520–0.905; *p* = 0.008), VTmax >6 s (OR, 1.02; 95% CI, 1.017–1.031; *p* = 0.014), VTmax >10 s (OR, 1.10; 95% CI, 1.067–1.374; *p* = 0.003), and VrCBF <30% (OR, 1.08; 95% CI, 1.010–1.170; *p* = 0.025) were identified as independent indicators of favorable prognosis (Table 3).

**Table 3.** **Multivariable analysis of imaging predictors of good outcome**.

	Poor outcome	
	OR (95% CI)	*p*-value
Baseline NIHSS score	1.18 (1.076–1.298)	<0.001*
Distal basilar artery	1.05 (0.123–8.917)	0.967
Vertebral arteries	1.27 (0.199–8.089)	0.801
Posterior cerebral artery	0.96 (0.131–6.518)	0.937
BATMAN score	0.73 (0.558–0.942)	0.016*
pc-CTA score	0.70 (0.446–1.102)	0.123
NCCT pc-ASPECTS	0.71 (0.345–1.452)	0.345
CTA-SI pc-ASPECTS	0.67 (0.396–1.127)	0.131
CBF pc-ASPECTS	0.62 (0.446–0.853)	0.003*
CBV pc-ASPECTS	0.25 (0.077–0.804)	0.002*
MTT pc-ASPECTS	0.69 (0.520–0.905)	0.008*
Tmax pc-ASPECTS	0.82 (0.632–1.056)	0.123
RAPID Tmax >6 s pc-ASPECTS	0.71 (0.387–1.265)	0.211
PMI	3.72 (1.264–10.962)	0.017*
CAPS	2.64 (1.700–4.097)	<0.001*
VTmax >6 s (mL)	1.02 (1.017–1.031)	0.014*
VTmax >10 s (mL)	1.10 (1.067–1.374)	0.003*
VrCBF <20% (mL)	1.08 (0.965–1.212)	0.177
VrCBF <30% (mL)	1.09 (1.010–1.170)	0.025*
	No surviving	
Baseline NIHSS score	1.15 (1.046–1.265)	0.004*
pc-CTA score	0.59 (0.386–0.903)	0.015*
PMI	1.85 (1.312–2.618)	<0.001*
CBF pc-ASPECTS	0.66 (0.490–0.898)	0.008*
CBV pc-ASPECTS	0.62 (0.449–0.879)	0.007*
MTT pc-ASPECTS	0.81 (0.632–1.034)	0.090
VTmax >6 s (mL)	1.01 (0.998–1.015)	0.133
VTmax >10 s (mL)	1.03 (1.004–1.056)	0.022*
VrCBF <30% (mL)	1.04 (0.993–1.084)	0.097
Cerebellar tonsillar hernia	0.17 (0.046–0.602)	0.006*
Symptomatic intracranial hemorrhage	0.37 (0.124–1.131)	0.082
AICA infarction	0.43 (0.131–1.404)	0.162
Midbrain infarction	0.19 (0.051–0.730)	0.015*

Tmax, time to maximum; CBV, cerebellar blood volume; pc-ASPECTS, posterior-circulation Acute Stroke Prognosis Early Computed Tomography Score; NCCT, non-contrast computer tomography. * indicates *p* < 0.05.

**Fig. 4.**
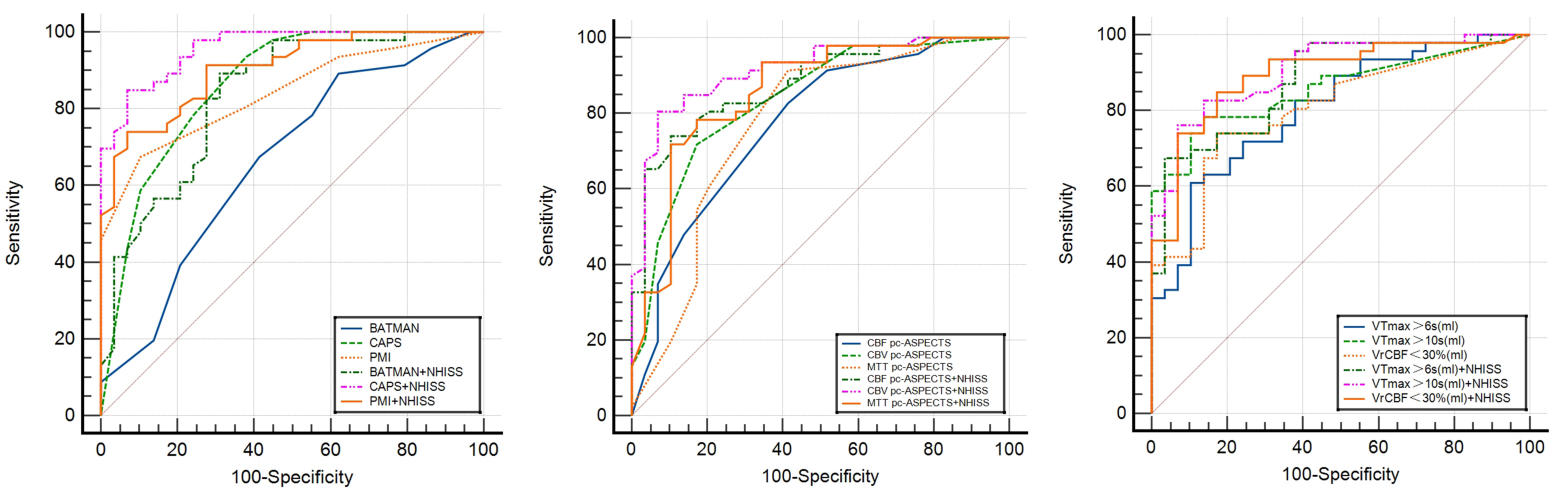
**Receiver operating characteristic (ROC) for imaging parameter and combined diagnosis prediction of good outcome in patients with basilar artery occlusion**.

In the ROC analyses, CAPS score (AUC, 0.862; 95% CI, 0.772–0.952; *p* < 0.001), PMI (AUC, 0.839; 95% CI, 0.751–0.926; *p* < 0.001), CBV pc-ASPECTS (AUC, 0.836; 95% CI, 0.742–0.930; *p* < 0.001), VTmax >6 s (AUC, 0.805; 95% CI, 0.706–0.904; *p* < 0.001), VTmax >10 s (AUC, 0.857; 95% CI, 0.774–0.941; *p* < 0.001), and VrCBF <30% (AUC, 0.804; 95% CI, 0.705–0.903; *p* < 0.001) showed outstanding efficacy in forecasting good outcomes at 90 days for individuals with acute BAO after EVT. CBF pc-ASPECTS (AUC, 0.771; 95% CI, 0.659–0.883; *p* < 0.001), MTT pc-ASPECTS (AUC, 0.768; 95% CI, 0.648–0.888; *p* < 0.001), and BATMAN scores (AUC, 0.664; 95% CI, 0.535–0.792; *p* = 0.017) were averaged (Table 4, Fig. 4).

**Table 4.** **ROC analyses of clinical and imaging parameters for good outcome prediction**.

	AUC (95% CI)	*p* value	Youden index	Cut-off value	Sensitivity	Specificity
CAPS	0.862 (0.772–0.952)	<0.001	0.556	2.5	87.00%	62.10%
PMI	0.839 (0.751–0.926)	<0.001	0.571	3.5	67.40%	89.70%
NIHSS	0.835 (0.742–0.929)	<0.001	0.538	21.0	84.80%	69.00%
VTmax >6 s	0.805 (0.706–0.904)	<0.001	0.506	87.5	60.90%	89.70%
VTmax >10 s	0.857 (0.774–0.941)	<0.001	0.645	4.1	78.30%	86.20%
VrCBF <30%	0.804 (0.705–0.903)	<0.001	0.567	3.2	73.90%	82.80%
CBV pc-ASPECTS	0.836 (0.742–0.930)	<0.001	0.545	7.5	71.70%	82.80%
CBF pc-ASPECTS	0.771 (0.659–0.883)	<0.001	0.412	6.5	82.60%	58.60%
MTT pc-ASPECTS	0.768 (0.648–0.888)	<0.001	0.499	6.5	91.30%	58.60%
BATMAN	0.664 (0.535–0.792)	0.017	0.270	5.5	89.10%	37.90%

Linear exclusion of the NIHSS score and imaging predictors were used for combined diagnosis. The AUC of combined CAPS and baseline NIHSS scores showed the best prediction performance (AUC, 0.958; 95% CI, 0.920–0.997, *p* < 0.001), with 83% sensitivity and 90% specificity. Moreover, combined CBV pc-ASPECTS (AUC, 0.914; 95% CI, 0.850–0.979; *p* < 0.001), PMI (AUC, 0.904; 95% CI, 0.839–0.969; *p* < 0.001), and VTmax >10 s (AUC, 0.906; 95% CI, 0.841–0.972; *p* < 0.001) also had excellent predictive accuracy. Admission NIHSS scores had different degrees and greater predictive accuracy of combined imaging diagnosis compared with single-factor diagnosis (Table 5).

**Table 5.** **ROC analyses of imaging parameters combined baseline NIHSS score for good outcome prediction**.

	AUC (95% CI)	*p* value	Youden index	Cut-off value	Sensitivity	Specificity
CAPS + NIHSS	0.958 (0.920–0.997)	<0.001	0.779	0.704	84.80%	91.30%
PMI + NIHSS	0.904 (0.839–0.969)	<0.001	0.670	0.753	73.90%	93.10%
VTmax >6 s + NIHSS	0.876 (0.797–0.954)	<0.001	0.618	0.769	65.20%	96.60%
VTmax >10 s + NIHSS	0.906 (0.841–0.972)	<0.001	0.688	0.601	82.60%	86.20%
VrCBF <30% + NIHSS	0.894 (0.820–0.968)	<0.001	0.676	0.577	84.80%	82.80%
CBV pc-ASPECTS + NIHSS	0.914 (0.850–0.979)	<0.001	0.735	0.688	80.40%	93.10%
CBF pc-ASPECTS + NIHSS	0.877 (0.800–0.954)	<0.001	0.636	0.728	73.90%	89.70%
MTT pc-ASPECTS + NIHSS	0.856 (0.764–0.948)	<0.001	0.614	0.755	71.70%	89.70%
BATMAN + NIHSS	0.833 (0.737–0.930)	<0.001	0.560	0.564	87.00%	69.00%

Additionally, we analyzed the predictive power for 90-day mortality. The multivariable binary logistic regression analyses included atrial fibrillation and smoking history (*p* < 0.1). Baseline NIHSS score, pc-CTA, PMI, CBF, CBV pc-ASPECTS, and VTmax >10 s were independently related to 90-day mortality. In ROC analysis, PMI (AUC, 0.795; 95% CI, 0.679–0.911; *p* < 0.001) and VTmax >10 s (AUC, 0.766; 95% CI, 0.651–0.881; *p* < 0.001) had a certain degree of predictive power for 90-day mortality (Table 4, Fig. 4).

## 4. Discussion

Our research findings indicate that CAPS, PMI, baseline NIHSS score, BATMAN score, CBF, CBV, MTT pc-ASPECTS, VTmax >6 s, VTmax >10 s, and VrCBF <30% were independent predictors of prognosis in patients with BAO undergoing EVT over 3 months. Moreover, CAPS, PMI, baseline NIHSS score, VTmax >10 s, CBF, CBV pc-ASPECTS, and pc-CTA score were independently associated with 90-day mortality.

Although CTP has a recognized worth in predicting the prognosis of stroke due to LVO in the anterior circulation, the value of CTP for stroke in the posterior circulation is poorly understood, and there is currently no consensus regarding this issue [31, 32]. Our findings indicate that the treatment and selection of patients with BAO can be influenced by the combination of clinical variables and baseline imaging.

Acute ischemic stroke resulting from BAO leads to considerable disability and mortality [33]. Previous randomized trials, such as BEST and BASIC, have failed to demonstrate an advantage in outcomes or death in patients with BAO undergoing EVT [13]. In the latest randomized trials—BAOCHE [10], BASILAR [9], and ATTENTION [8]—EVT showed effectiveness in improving outcomes for patients with BAO in comparison to acknowledged medical therapy alone. The BAOCHE trial included pc-ASPECTS for the first time and suggested that preoperative perfusion images might benefit patients with BAO undergoing EVT. Previous studies have shown that bridging thrombolysis successfully improves survival rates of BAO and that intravenous thrombolysis can promote local thrombolysis, achieve reperfusion, and clear distal thrombus [22, 34, 35]. Despite the absence of a statistically significant difference between survival and non-survival groups, more patients survived intravenous thrombolysis, indicating that intravenous thrombolysis or bridging thrombolysis may effectively decrease the mortality rates at 90 days.

Parsons* et al*. [36] and Da Ros* et al*. [37] showed that the pc-CTA score was a good predictor of preoperative outcomes and that pc-ASPECTS of CBV maps improved predictive power for patients with highly negative functional outcomes. Simultaneously, poor outcomes in 60 patients with BAO can be independently predicted by CBV pc-ASPECTS ≤8. The performance of our sample on CBV pc-ASPECTS aligns with these results. However, the difference is that pc-CTA or pc-CS performs unsatisfactorily, which may be explained by the fact that all patients included in our study underwent early endovascular therapy post-examination, hence mitigating the influence of collateral compensation on prognosis. The optimal cut-off value for pc-ASPECTS currently applied to posterior circulation occlusion is still controversial [11]. In our study, the cut-off values of pc-ASPECTS in CBV, CBF, and MTT maps were 7, 6, and 6, respectively, higher than what was known in the BASILAR. Which may be a further indication that the pc-ASPECTS scores assessed on CTP images are more accurate than those assessed on NCCT, where the posterior fossa brain structures are challenging to display clearly [32], and by ischemic changes in the brainstem in most patients in our sample, which may account for a large proportion of the scores.

The pc-ASPECTS has the advantage of being quick and simple when facing emergencies. In our study, CBV pc-ASPECTS was better for identifying negative patients, whereas CBF pc-ASPECTS demonstrated greater sensitivity in diagnosing positive patients. However, the median pc-ASPECTS of Tmax maps was significantly lower than that of CBV and CBF, and the median of MTT maps was also low. In healthy individuals, blood flow velocity in the posterior circulation is slower than that in the anterior circulation. Therefore, the application value of Tmax and MTT maps in the posterior circulation may not be as accurate as that of CBV and CBF maps, and there may be some defects in distinguishing the severity of the infarction area. The lack of objectivity is also a problem.

The size of the infarct is generally considered to have an important influence on clinical prognosis. Puetz* et al*. [29] investigated the correlation between hypoperfusion volume and the prognosis in patients with BAO by manually selecting the maximum extent of lesions delineated by the ROI. As of yet, no comprehensive, randomized trial of patients with BAO undergoing EVT has included low perfusion volume as an entry criterion for screening patients. Although the study confirmed that it has a good predictive value, higher than pc-ASPECTS, manual selection is time-consuming and observer-dependent, much like the pc-ASPECTS score. Therefore, to overcome the limitations of manual methods and explore more objective methods, our study selected Siemens post-processing workstation Syngo.via to define hypoperfusion volumes by setting different CT thresholds, a fully automated method. We found that VTmax >10 s, VTmax >6 s, and VrCBF <30% were good independent predictors. VTmax >10 s performed better in our sample and was perhaps symmetrically associated with brain involvement due to posterior circulation ischemia, while VrCBF <30% focused on bilateral brain involvement, suggesting that the total amount of hypoperfusion due to posterior circulation ischemia may be more relevant to patient prognosis. At the same time, we found that VTmax >10 s had some value in predicting 90-day mortality, which was slightly lower than PMI.

The ischemic penumbra and core can predict the prognosis of AC-LVO, whereas the location of infarction may be more important than the extent of the ischemic core in BAO [38]. Cereda* et al*. [21] defined pontine, midbrain, and other regional CAPS according to Tmax >10 s maps, which was found to be an excellent predictor. These findings were consistent with those of our study, and it is highly valuable for testing positive patients. CAPS could be considered for screening patients who have achieved effective reperfusion for reference. At the same time, we found that CAPS combined with the baseline NIHSS score had better predictive power, which suggests that CAPS could be a good complementary tool to NIHSS. The independent predictive power of CAPS may need to be verified in larger prospective trials in the future, and better performance could be expected by manually delineating ischemic core regions or setting different specific thresholds. In addition to the strong correlation of PMI in predicting clinical outcomes in our study, PMI also had excellent predictive power in predicting 90-day mortality, which may indicate that the involvement of the infarction site has a significant influence on the functional outcome of reperfusion in patients; for example, brainstem involvement in basic vital activities such as heartbeat and respiration [4], if it is once involved or more, is likely to cause devastating clinical outcomes after EVT. CAPS and PMI are scores designed to focus on the patient’s infarction site. In particular, CAPS scores can accurately identify patients with small ischemic core areas, and their predictive power in our sample is strong and exceeds pc-ASPECTS of any image, which may verify that the importance of the infarction site in BAO exceeds the importance of the extent of the infarction core. While CAPS and PMI focus on the specific site of infarction, they occupy a relatively limited area of posterior circulation ischemia, which may be a limitation of these methods.

The Revascularization in Ischemic Stroke Patients (REVASK) [39] indicated that a lower baseline NIHSS score was an independent predictor of good prognosis at 90 days. In comparison, a higher baseline NIHSS score was an independent predictor of mortality. Our findings align with prior research. Recent studies have shown that NIHSS scores, regardless of their severity, are a useful tool for guiding treatment options and clinical outcomes in patients with BAO [10, 40]. Interestingly, after collinearity elimination of baseline NIHSS scores and various other factors, we found that the prediction accuracy of CAPS, PMI, CBV, CBF, MTT pc-ASPECTS, Tmax >10 s, >6 s, and rCBF <30% volume was improved to different degrees compared with that of a single prediction. Moreover, the combined AUC value of CAPS, CBV pc-SAPECTS, PMI, and VTmax >10 s diagnosis was excellent (AUC >0.9), with high diagnostic power. In the future, we aspire to acquire larger datasets for validation and formulate clinically pertinent simplified calculation models that integrate clinical and imaging data, thereby enhancing the prediction accuracy for patients with BAO.

There were several limitations in our study. First, this was a retrospective analysis with a limited sample size. Thus, further research with bigger cohorts is necessary to corroborate our results. Moreover, the patients we studied all came from the same comprehensive stroke center; therefore, the prevalence may be limited. Finally, our study’s CT perfusion software package is not well established for use in posterior circulation stroke, and the single-phase CTA we used may have limitations such as underestimating collateral circulation.

## 5. Conclusion

In conclusion, CAPS, PMI, CBV pc-ASPECTS, Tmax >10 s, Tmax >6 s, and rCBF <30% volume can be used to predict prognosis at 90 days in patients with BAO undergoing EVT. Combined diagnosis with baseline NIHSS score can improve the predictive accuracy of prognosis and provide a reference for clinical intervention measures, especially for prognosis judgment of EVT recanalization patients, holding certain clinical practical significance and potentially offering more information for future research.

## Availability of Data and Materials

The datasets used and analyzed during the current study are available from the corresponding author on reasonable request.
